# Influence of greater trochanteric bone density and three-dimensional morphology on perioperative greater trochanteric fracture following total hip arthroplasty via an anterolateral approach

**DOI:** 10.1186/s12891-023-06988-5

**Published:** 2023-10-31

**Authors:** Daisuke Inoue, Tamon Kabata, Yoshitomo Kajino, Yuki Yamamuro, Atsushi Taninaka, Tomoyuki Kataoka, Yoshitomo Saiki, Yu Yanagi, Musashi Ima, Takahiro Iyobe, Hiroyuki Tsuchiya

**Affiliations:** Department of Orthopedic Surgery, Graduate School of Medical Science, Kanazawa University, 13-1 Takaramachi, Kanazawa, Ishikawa, 920-8641 Japan

**Keywords:** Total hip arthroplasty, Bone mineral density, Anterolateral approach, Perioperative greater trochanteric fracture

## Abstract

**Background:**

Perioperative greater trochanteric fracture following total hip arthroplasty (THA) using the anterolateral approach is a recognized perioperative complication. There was no previous study to determine the relationship between bone mineral density (BMD) and three-dimensional greater trochanter morphology for perioperative greater trochanter fractures. The purpose of this study is to identify the influence of greater trochanteric bone density and three-dimensional morphology on perioperative greater trochanteric fracture following THA using the anterolateral approach.

**Methods:**

We investigated 209 hips done primary THA using the anterolateral approach and preoperative BMD test for the proximal femoral bone with a minimum of 6 months follow-up. We picked up all patients who had perioperative greater trochanteric fractures. Multivariate analysis was done in order to investigate the influence of the greater trochanter young adult mean (YAM) and three-dimensional morphology on perioperative greater trochanteric fractures.

**Results:**

There were 10 joints (10/209, 4.8%) with perioperative greater trochanteric fractures. Osteosynthesis was required only in one joint (1/209, 0.5%) because the bone fragments were significantly displaced proximally by the gluteus medius. Multivariate analysis showed the combination of Type B femoral shape (in cases where the top of the great trochanter was inside the longitudinal central axis of the planned femoral stem in computed tomography (CT)- based three-dimensional templating) and a YAM of < 80% was the only risk factor for fracture.

**Conclusions:**

The preoperative greater trochanter BMD test (YAM < 80%) and three-dimensional femoral morphology (Type B femoral shape) provide useful information to mitigate the occurrence of perioperative greater trochanter fractures associated with THA using the anterolateral approach.

## Introduction

Recently, minimally invasive surgery using the direct anterior and anterolateral approach for total hip arthroplasty (THA) has often been adopted by orthopedic surgeons [1]. However, a previous study showed that perioperative fracture occurred more frequently in the anterior than in the posterolateral approach [2]. Perioperative greater trochanter fracture is known to be a typical perioperative complication in direct anterior and anterolateral approach. This fracture is not rare since previous reports have indicated an incidence rate of 2–30% [3–6]. In comparison to intraoperative periprosthetic femoral fractures and periprosthetic joint infections, a greater trochanteric fracture may be categorize as a minor postoperative complication. However, a Trendelenburg gait and residual limping may result from the large bone fragment being proximally displaced by the gluteus medius. Therefore, it is mandatory to avoid a perioperative greater trochanter fracture during primary THA.

Previous studies have determined risk factors for perioperative greater trochanter fracture using direct anterior and anterolateral approach [3–5]. Recent study has revealed a higher risk for perioperative greater trochanteric fractures in femoral geometry where the top of greater trochanter was inside the longitudinal central axis of the planned femoral stem in Computed Tomography (CT)-based preoperative planning [6]. This report was the first study to identify the risk factors of perioperative greater trochanteric fractures in preoperative three-dimensional template. Meanwhile, it is well known that perioperative femoral fractures are greatly influenced by patient's bone quality [3–8]. However, there was no previous study investigating the interrelationship between bone mineral density (BMD) and three-dimensional greater trochanter morphology for perioperative greater trochanter fractures following THA using the anterolateral approach.

Hence, the purpose of this study was to clarify the influence of greater trochanter bone density and three-dimensional morphology on perioperative greater trochanteric fracture following THA using the anterolateral approach.

## Materials and methods

This investigational protocol was conducted with approval from the institutional ethics committee. This retrospective study included a consecutive series of patients with primary unilateral THA using an anterolateral approach between January 1, 2016, and December 31, 2020. All operations were done by the senior surgeon (T.K). The patients who were followed up for minimum of 6 months were included in this study. Also, patients who underwent a bone mineral density (BMD) test for the proximal femoral bone using dual-energy X-ray absorptiometry (DEX) preoperatively were retrieved from this retrospective cohort. The patient who was followed up for less than 6 months was excluded from this study. After all candidates who met the inclusion criteria were recruited, candidates’ demographic data (including age, sex, body mass index (BMI), etiology of the hip disorder, and type of femoral implant used) were retrieved from our institutional medical electronic database.

**Fig. 1:**
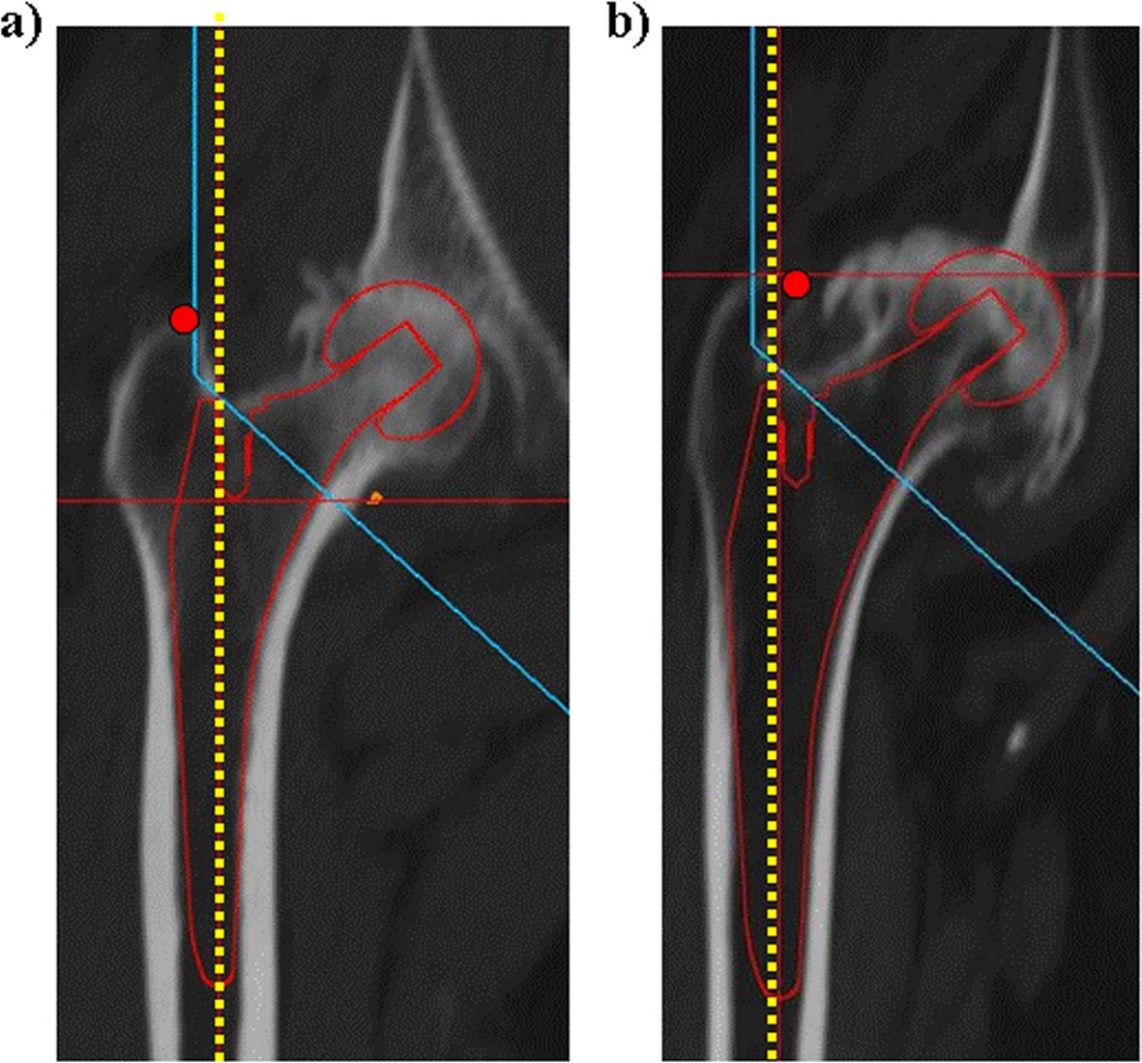
Relative relationship between the top of the greater trochanter and planned proximal stem in the femur. **a** Type A: The top of the great trochanter is outside the longitudinal central axis of the planned femoral stem. **b** Type B: The top of the greater trochanter is inside the longitudinal central axis of the planned femoral stem

Preoperative hip radiographs were used to calculate the canal flare index (CFI) and Dorr classification, according to previous papers [9, 10]. Next, regarding with femoral three-dimensional morphology, the relationship between the greater trochanter and planned femoral stem was evaluated by the positional relationship between the top of the greater trochanter and the femoral stem longitudinal axis in three-dimensional preoperative planning using ZedHip (Lexi Co., Tokyo, Japan), as described in our previous papers [6, 11]. Type A was defined as a case in which the top of the greater trochanter was outside the longitudinal central axis of the planned femoral stem, and Type B was defined as a case in which the top of the greater trochanter was inside the longitudinal central axis of the planned femoral stem (Fig. 1) [6]. Data on BMD was collected in the proximal femoral bone using DEX to assess for greater trochanter bone quality. Data on the young adult mean (YAM) for the proximal femoral bone was also retrieved using DEX.

### Assessment

We retrieved all cases with perioperative greater trochanteric fractures regardless of the need for osteosynthesis due to proximal displacement of the bone fragment by the gluteus medius. Subsequently, we compared demographic data and femoral geometry data between the fracture and non-fracture groups. Furthermore, multivariate analysis was done to identify influence of greater trochanteric bone density and three-dimensional morphology of perioperative greater trochanteric fractures on femoral geometry data.

### Statistical analysis

We used Easy R (EZR) (Saitama Medical Centre, Jichi Medical University, Japan), which is a graphical user interface for R (R Foundation for Statistical Computing, Austria) for the statistical analysis [12]. We compared patient demographic and femoral geometry data between two groups using Mann–Whitney U test for continuous variables, and the Fisher's exact probability test for categorical variables. Multivariate analysis was performed using a logistic regression model. A *P* value < 0.05 was considered statistically significant.

## Results

A total of 353 primary unilateral THAs via the anterolateral approach were performed during study period. Five THAs were excluded because follow-up period was within 6 months. Preoperative BMD testing was not performed for 139 THAs. Finally, 209 THAs were enrolled in this study.

There were 10 joints (10/209, 4.8%) in perioperative greater trochanteric fractures. Cementless stem was used in 9 of 10 fracture cases, whereas cemented stem was used in 1 of 10 cases. Concerning the time of detection of perioperative greater trochanteric fractures, six occurred during the intraoperative procedure, including two, three, and one cases of femoral rasping, femoral retraction, and femoral neck cutting, respectively. On hip radiography, three cases were detected at 1 week postoperatively, and one case was detected at 2 weeks postoperatively, although a greater trochanteric fracture was not detected on postoperative hip radiography. We did osteosynthesis in only one case of all joints with perioperative greater trochanteric fractures because the bone fragments were significantly displaced proximally by the gluteus medius.

There was no statistical difference in the demographic data, such as age, sex, BMI, and etiology of the hip disorder, between the enrolled and excluded patients. The results of demographic data in all candidates are shown in Table 1. All perioperative greater trochanteric fractures occurred in women, although there was no statistical difference between the two groups. There were no statistical differences in age, BMI, and etiology of the hip disorder. In the case of femoral implants, almost 95% were either tapered wedges or curved short stems. There were 125 cases in Tapered wedge stems, 74 cases in curved short stems, and 10 cases including cemented, full hydroxyapatite, or short anatomical stems. The incidence rate of perioperative greater trochanteric fractures in the tapered wedge stem was 5.6% (7/125) and that in the curved short stems was 2.6% (2/76). There was no statistical difference between the two groups (*P* = 0.49).

**Table 1:** Demographic data of enrolled patients

	**Fracture group** **(*n* = 10)**	**No fracture group** **(*n* = 199)**	***P* value**
**Age**	64.8 ± 6.3	63.1 ± 10.7	0.46
**Male/Female**	0/10	30/169	0.36
**BMI**	24.6 ± 4.1	24.3 ± 5.1	0.73
**Disorder**			
OA	9 (90.0%)	172 (86.4%)	1.0
ONFH	0	26 (13.1%)	
RA	1 (10.0%)	1 (0.5%)	
**Used femoral implant**			
Tapered wedge stem	7 (70.0%)	118 (59.3%)	0.49
Curved short stem	2 (20.0%)	72 (36.2%)	
Other type of stem	1 (10.0%)	9 (4.5%)	

Age and BMI are shown in mean ± S.D.

*BMI* Body mass index, *ONFH* Osteonecrosis of femoral head

The femoral geometry data are shown in Table 2. There was no statistical difference in CFI and Dorr classification between the two groups. Meanwhile, there was a statistically significant difference in Type B femoral geometry (*P* = 0.009). Furthermore, regarding the YAM with preoperative DEX test, the fracture group showed less YAM in the greater trochanter and femoral neck in comparison with the non-fracture group. Based on these data, when femoral geometry was divided into two groups (A and B) and YAM of the greater trochanter was divided into two groups (> 80% and < 80%). Using multivariate analysis, the combination of Type B femoral geometry and YAM < 80% was an independent risk factor of perioperative greater trochanteric fracture (odds ratio 33.7; 95% confidence interval, 5.13–222; *P* < 0.001) (Table 3).

**Table 2:** Femoral geometry data of enrolled patients

	**Fracture group** **(*n* = 10)**	**No fracture group** **(*n* = 199)**	***P* value**
**CFI**	3.5 ± 0.1	3.7 ± 0.3	0.13
**Dorr classification**			
Type A	0	14	0.36
Type B	10	169	
Type C	0	16	
**Relative relationship between the top of great trochanter and planned proximal stem**			
Type A	6 (60.0%)	183 (92.3%)	0.009
Type B	4 (40.0%)	16 (7.7%)	
**Young adult mean (YAM)**			
Greater trochanter	80.5 ± 7.0	91.5 ± 20.4	0.01
Femoral neck	83.0 ± 10.0	95.1 ± 16.8	0.001

CFI are shown in mean ± S.D.

*CFI* Canal flare index

**Table 3:** Result of multivariate analysis for the risk of perioperative greater trochanteric fracture

Variables	B	S.E	Odds ratio (95% CI)	*P* value
Type A and YAM ≧ 80	-	-	-	-
Type A and YAM < 80	0.320	0.881	1.38 (0.25–7.76)	0.72
Type B and YAM ≧ 80	0.954	1.155	2.60 (0.27–25.0)	0.41
Type B and YAM < 80	3.519	0.961	33.7 (5.13–222)	< 0.001

*YAM* Young adult mean B, regression coefficient, *SE* Standard error, *95% CI* 95% confidence interval

## Discussion

Previous reports have investigated the risk factors of perioperative greater trochanteric fractures associated with the direct anterior or anterolateral approach. With respect to sex, previous some papers reported that female tended to occur perioperative greater trochanteric fracture [13–15]. Meanwhile, regarding femoral geometry, three papers identified risk factors for perioperative greater trochanteric fractures using a direct anterior or anterolateral approach. Homma et al. reported that the greater size of the posterior aspect of the greater trochanter on axial CT slices tend to occur perioperative greater trochanter fracture [3]. Hartford et al. determined that lower femoral neck cut ratio and greater Dorr ratio were statistically significant indicators associated with perioperative greater trochanteric fractures [4]. Recently, Inoue et al. identified Type B femoral geometry in three-dimensional preoperative planning as an important risk factor in perioperative greater trochanteric fractures [6]. Since healthy life expectancy has increased in recent years, it is no longer uncommon for elderly patients with low bone density to undergo primary THA. Hence, it is becoming increasingly important to confirm the preoperative bone quality of patients undergoing primary THA because low bone density has been known to be an important risk factor in perioperative femoral fracture during primary THA. A previous multicenter study conducted in eight Danish centers indicated that osteoporosis was an independent risk factor for periprosthetic femoral fracture (*RR* = 1.6, CI: 1.1–2.2) within 90 days after operation [8]. Berliner et al. showed that patients with osteoporosis aged > 70 years tended to have periprosthetic femoral fractures during THA via the direct anterior approach [16]. In addition, a recent study showed that FRAX scores were significantly higher in patients with periprosthetic femoral fracture than in those with no periprosthetic femoral fracture [17]. Considering these previous reports, it seems that assessing the preoperative bone density test is important because the risk of perioperative femoral fracture tends to increase when osteoporosis is present on the preoperative DXA test. However, no previous study has explored the interrelationship between BMD and the three-dimensional morphology of the greater trochanter in perioperative greater trochanter fractures via the anterolateral approach. Hence, we investigated the influence of greater trochanter bone density and three-dimensional morphology on perioperative greater trochanteric fracture following THA using the anterolateral approach.

To the best of my knowledge, our study is the first to identify the influence of greater trochanter bone density and three-dimensional morphology on perioperative greater trochanteric fracture of THA using the anterolateral approach. In our study, multivariate analysis revealed that the combination of Type B femoral geometry and a YAM of < 80% was the only independent risk factor for perioperative greater trochanteric fracture. These results show that the preoperative DXA test and CT-based three-dimensional preoperative templating provide useful information which can be used to predict and mitigate the risk of perioperative greater trochanter fractures during primary THA using the anterolateral approach prior to the operation. However, there was a concern to perform the BMD test on all patients undergoing THA because of the cost of the procedure. Therefore, we considered that the BMD test could be performed in cases of Type B three-dimensional morphology of the greater trochanter to determine the perioperative greater trochanter fracture preoperatively. The adequate lateral elevation of proximal femur was necessary for safety femoral rasping in order to prevent perioperative greater trochanter fractures. Hence, in cases known preoperatively to be prone to this fracture, we feel that releasing the conjoint tendon may contribute to mitigate the risk of perioperative greater trochanter fracture by reducing the traction force on the greater trochanter because most perioperative greater trochanter fractures occur due to the traction force of the conjoint tendon, as mentioned in some previous papers [18, 19].

There are some limitations to this study that should be considered when interpreting the findings. First, this single center study was retrospective in nature, and the total number of candidates was relatively small because some candidates did not undergo preoperative DXA; however, a power analysis showed that if we were to assume the incidence rate of perioperative greater trochanteric fractures in previous studies with an α-error of 0.05 and power of 80%, we would need approximately 145 cases in this study [3–6]. Therefore, this study was not underpowered. Second, due to its retrospective nature, some kinds of femoral stems were used in this study. However, we feel that the femoral stem used in this study did not have a large impact on the analysis because either tapered wedges or curved short stems were used in 95% of all THAs. Furthermore, there was no statistical difference among the groups (*P* = 0.49). However, the use of cementless stem for patients with osteoporosis may be a risk factor of perioperative greater trochanteric fracture because cementless stems have higher rates of periprosthetic femoral fractures. Our study included a small number of cases using cemented stems. Hence, this was a limitation of the study because of the study’s retrospective nature and small number of candidates using cemented stems. We believe that this point needs to be clarified in a future multicenter study or a systematic review. Third, the learning curve for THA via anterolateral approach may influence the incidence rate of perioperative greater trochanteric fractures. However, a recent previous paper showed the incidence rate of perioperative greater trochanteric fractures did not decrease with surgeon experiences. Furthermore, all procedures in this study were done by the experienced an attending doctor. Hence, we feel the learning curve for THA via anterolateral approach may have not a significant influence on the result of this study.

In conclusion, there were 10 joints (10/209, 4.8%) with perioperative greater trochanteric fractures. Osteosynthesis was necessary only in one joint (1/209, 0.5%). Surgeons should note that the incidence rate of this fracture is not low. This study identified that the combination of a greater trochanter bone density of < 80% with Type B femoral geometry in CT-based preoperative three-dimensional templating posed the highest risk for perioperative greater trochanteric fractures during THA using anterolateral approach. The preoperative greater trochanter BMD test and three-dimensional femoral morphology provide useful information to mitigate the occurrence of perioperative greater trochanter fractures associated with THA via the anterolateral approach.

## Data Availability

The datasets used and/or analysed during the current study are available from the corresponding author on reasonable request.
